# Epidemics on the move: Climate change and infectious disease

**DOI:** 10.1371/journal.pbio.3001013

**Published:** 2020-11-24

**Authors:** Matthew B. Thomas

**Affiliations:** York Environmental Sustainability Institute and Department of Biology, University of York, York, United Kingdom

Understanding the factors that govern the dynamics and distribution of animal and plant parasites and pathogens has taken on a new urgency in the face of global climate change. Many disease-causing organisms are strongly influenced by environmental factors such as temperature, rainfall and humidity, which are in turn influenced by climate change. For this reason it is widely expected that climate change will affect infectious disease patterns. Much of the early research on climate change and infectious disease emphasized the potential for increases in disease risk under future climate scenarios, with range expansion or changes in seasonality anticipated to lead to net increases in transmission [1]. This research led to a sense that a “warmer world would be a sicker world.” Recent research has provided a more nuanced perspective, highlighting the potential for a “two-tailed” response, meaning that shifts in climate could drive conditions towards the optimum for transmission in some areas while pushing conditions away from the optimum in others [2,3]. Indeed, if parasites and pathogens follow the patterns predicted for other taxa, it is reasonable to expect that some diseases will adapt to changing environmental conditions and potentially increase in prevalence, whereas others will suffer negative consequences leading to range contractions and even local extinctions. Yet researchers face the considerable challenge of determining which outcome will apply to which diseases, and where and when predicted changes may occur.

This issue of *PLOS Biology* features four Essays that attempt to unravel some of this complexity in the collection “Epidemics on the move: climate change and infectious disease.” We deliberately sought papers covering a diversity of disease systems, but as a common theme we asked the authors to highlight some key recommendations and approaches for further research.

The paper by Jason Rohr and Jeremy Cohen takes an ecological perspective to explore factors and mechanisms shaping the possible effects of climate change on disease in a range of terrestrial and aquatic systems [4]. Jeb Byers takes a similar approach to explore diseases in near-shore and estuarine systems [5]. A feature of both papers is that they highlight the importance of thermal ecology, since even small changes in temperature can have strong non-linear effects on the outcome of ectotherm host-parasite and host-pathogen interactions. Jeremy Burdon and Jiasui Zhan also emphasize the need to improve mechanistic understanding of the influence of abiotic factors for plant pathogens, but highlight additionally how patterns might play out differently in simplified, managed (that is, agricultural) ecosystems compared to more complex natural systems [6]. Rachel Lowe and colleagues build on the role of context further, and move beyond biology to consider more explicitly the issues of management to examine not only how climate change might increase the threat of vector-borne diseases but also what needs to be done in a vulnerable setting, such as the Caribbean, to build a resilient system and reduce the risks [7].

It is impossible to provide an exhaustive treatment of diseases, ecosystems, or mechanisms in a small collection of papers. You will find limited coverage of fungal diseases, for example, yet it is possible that climate change may select for new fungal pathogens, affecting people, animals and plants, as species with pathogenic potential adapt to higher temperatures [8]. This general question of evolutionary change and potential for thermal adaptation represents one of the big unknowns highlighted across the papers in this collection. The authors identify a number of mechanisms and approaches to help better understand the effects of climatic factors on coupled host-pathogen or host-parasite interactions. But they acknowledge that we have a long way to go to gain a better understanding of how these interactions will play out over longer time scales and when these pairwise interactions are embedded in more complex ecological communities. Additionally, the papers emphasize how other drivers of environmental change, such as land use changes, urbanization, biodiversity loss, and invasive species, could interact with climate change to potentially amplify or reduce disease risk. Furthermore, ultimate risk and impact of certain diseases is strongly influenced by the capacity for disease control. If a disease can be controlled effectively, the consequence of climate (and climate change) could largely be mitigated, but where control is difficult or impossible, the consequences could be substantial. This issue highlights the importance of context; in the case of diseases affecting humans, the vulnerability to climate change can depend as much on local capacity, socio-economics and political factors as on ecological factors [9,10].

Clearly, the overall influence of climate change on disease is complex. However, if there was ever any doubt about how disruptive emerging diseases can be, the COVID-19 pandemic has underscored the potential for infectious disease to wreak total havoc. Accordingly, increasing understanding of how climate change can affect the dynamics and distribution of infectious disease has never been more relevant. We hope this collection helps guide and stimulate further research towards this goal.
